# No difference in prevalence of spine and hip pain in young Elite skiers

**DOI:** 10.1007/s00167-017-4733-1

**Published:** 2017-12-04

**Authors:** Carl Todd, Anna Swärd Aminoff, Cecilia Agnvall, Olof Thoreson, Leif Swärd, Jon Karlsson, Adad Baranto

**Affiliations:** 1 Department of Orthopaedics, Institute of Clinical Sciences at Sahlgrenska Academy, University of Gothenburg and Sahlgrenska University Hospital, Göthenburg, Sweden; 2 Sportsmedicine Åre and Åre Ski High School, Göthenburg, Sweden; 3 The Carl Todd Clinic, 5 Pickwick Park, Park Lane, Corsham, SN13 0HN UK

**Keywords:** Athletes, Back, Hip, Pain, Skiers, Spine

## Abstract

**Purpose:**

To investigate the prevalence between back and hip pain in young Elite skiers.

**Methods:**

Sample group (*n* = 102), consisted of young Elite skiers (*n* = 75) and age-matched non-athletes (*n* = 27), all completed a three-part back and hip pain questionnaire, Oswestry Disability Index and EuroQoL to evaluate general health, activity level, back and hip pain prevalence.

**Results:**

No significant differences were shown for lifetime prevalence of back pain in the skiers (50%) compared with controls (44%) (n.s.). Duration of back pain for the skiers showed (30%) > 1 year, whilst (46%) > 5 years. A significant difference was shown with increased Visual Analogue Scale back pain levels for skiers 5.3 (SD 3.1) compared with controls 2.4 (SD 1.9, *p* = 0.025). No significant differences were shown for lifetime prevalence of hip pain in skiers (21%) compared with controls (8%) (n.s.).

**Conclusion:**

Young Elite skiers are shown not to have increased lifetime prevalence for back and hip pain compared with a non-athletic control group.

**Level of evidence:**

II.

## Introduction

The prevalence of low back pain (LBP) has been shown to be common in young elite athletes compared with age-matched non-athletes [2, 5–7, 18, 20, 22, 27, 33–36]. Increased levels of physical activity and high axial spinal loading have been suggested as risk factors for LBP prevalence in elite sports [33]. LBP in the absence of serious medical or psychological conditions has previously been classified as either (1) mobility impairment in the thoracic, lumbar and/or sacroiliac regions, (2) referred or radiating pain to the lower extremities, and (3) as generalized pain [11]. Mobility impairment and referred or radiating pain is normally related to the diagnosis of a particular patho-anatomical structure such as disc herniation and may be classified as specific LBP. Non-specific or generalized pain is used for those patients that fail to have a precise patho-anatomical diagnosis. It is estimated that approximately 85% of LBP patients are classified as non-specific [25].

Similarly a high prevalence of hip pain has been reported in young elite athletes [18, 26]. Hip pain may include extra- and intra-articular pathology or a combination of both that results in clinical signs and symptoms such as buttock, lateral, anterior and posterior thigh, groin, knee and LBP [13, 14]. Extra-articular hip pain may relate to soft tissue pathologies such as tendinopathy, bursitis, hernia and muscle strain [13]. Moreover, intra-articular hip joint pain is also shown to be common [30], with Femoroacetabular impingement syndrome (FAI) reported as a frequent cause of pain and shown to have a prevalence of up to 89% in elite athletes [9, 10, 28, 30–32].

Young Elite Alpine skiers have been shown to at risk of developing LBP [7, 27, 33]. Moreover, participation in elite skiing during adolescence has been shown to result in different spinal sagittal alignments and increased spinal and hip joint radiological changes compared with non-athletes [36–38, 41]. It has been suggested that sports requiring repetitive and excessive hip joint mobility may become more susceptible to LBP [40]. One explanation for this may be the close anatomical relationship between the hip joint and the spino-pelvic complex [15]. Moreover, postures attributed to the biomechanics of Alpine skiing such as spinal, hip and knee joint flexion that may aid with the absorption of shock [16, 21], may actually contribute to increased overload upon the axial spine and hip joint [33].

Therefore, it is reasonable to suggest that young Elite skiers are at a greater risk of developing increased levels of back and hip pain compared to non-athletes. Further studies may help to provide an evidence-based approach to inform coaching and training decisions to monitor the skiers loading in preparation for competition. Therefore, the purpose of the present study is to evaluate the relationship between the lifetime prevalence of back pain and hip pain among young Elite skiers and also to correlate these findings and compare them to a non-athletic age-matched control group. The hypothesis of the present study is to show that there is increased lifetime prevalence of back pain and hip pain among young Elite skiers compared with a non-athletic control group.

## Materials and methods

The sample group (*n* = 102) consisted of young Elite Alpine skiers (*n* = 75) and an age-matched non-athletic population (*n* = 27). Inclusion criteria for the skiers group were training and competing at elite level, High School grades 1–4, between 16 and 20 years of age, who were recruited from the Åre High School Ski Academy, Sweden. The control group was recruited from first year High School pupils. All participants were invited to partake in this prospective study after a short presentation about the project by two of the authors. The questionnaires were completed at the Elite Ski School in Åre and the Östersund Hospital in Östersund, Sweden.

Participants (skiers and controls) were excluded if they had a history of previous surgery to the lumbar spine, pelvis or hip joint or a history of systemic pathology including inflammatory arthritis or pelvic inflammatory disorders and pregnancy. Inclusion criterion for the control group was that they had not previously or at present participated in any organized sporting activities or trained for more than 2 h per week. The demographic characteristics of the full cohort are presented in Table 1.

**Table 1:** Baseline characteristics for all subjects stratified by group

	All subjects (*n* = 102)	Skiers (*n* = 75)	Controls (*n* = 27)	*p* value
Age (years)	18	18.2 (1.1)	16.4 (0.6)	< 0.001^b^
Gender female/male (%)	52/48	47/53	67/33	n.s.^a^
Height (cm)	173	174 (8.2)	172 (8.6)	n.s.^b^
Weight (kg)	69	70 (9.1)	67 (17.9)	n.s.^b^
Body mass index (kg/m2)	22.9	22.9 (2.2)	22.7 (5.3)	n.s.^b^

Values are mean and (standard deviation; SD) unless specified. *BMI* body mass index

^a^Chi-square test

^b^Independent sample *t* test

The back and hip questionnaire was developed and by Swärd et al. [34] and Baranto et al. [6] and has been widely used in other studies [36, 41]. It focuses on back and hip pain relating to previous and present levels of pain, local or radiating, activity level, etc., in young athletes. Back and hip questions are designed to evaluate the nature, onset and duration of pain, if the pain was correlated to exercise or competition, and if any movements aggravated or relieved the pain. Back and hip pain was self-assessed and graded moderate or severe. Classification of moderate levels was determined if the daily living, work, training or competition was not affected by back pain. Classification of severe levels was determined if the back or hip pain influenced daily living, work, training or competition. The location and type of pain was investigated by using the Visual Analogue Scale (VAS). This was self-assessed by the patient on a 10-cm vertical scale marked 0–100, with 0 equating to the worst imaginable health and 100 the best imaginable health.

The ODI questionnaire evaluates back pain in relation to daily life activities where pain intensity and ability is rated subjectively [12]. Six answer scores, 0–5 where 5 is the greatest disability, are presented to each of the 10 questions. The scores are then summed up, doubled and converted to percentage. The results are interpreted as minimal disability 0–19%, moderate 20–39%, severe 40–59%, crippling 60–79%, and bed bound 80–100%.

The EQ-5D (The EuroQol Group 1990) is a practical way to measure health of a population and differences within subgroups of the population [19]. Measurement includes assessment of daily life activity, ability to move, self-care, pain and anxiety levels. The questionnaire is rated levels 1–3, ranging from level 1 indicating no problems, level 2 indicating some problems and level 3 indicating lots of problems. The present study was approved by the Regional Ethical Review Board in Gothenburg at The Sahlgrenska Academy, Gothenburg University, Gothenburg, Sweden (ID number: 692-13).

### Statistical analysis

Data were analysed using IBM SPSS Statistics for Windows, Version 22.0. Armonk, NY: IBM Corp. The description of data was expressed in terms of the mean and standard deviation (SD), median and range including frequencies and percentages were appropriate. Pearson Chi-square test was performed to compare the distribution of back pain between groups, as Fisher’s exact test was performed to compare the distribution of hip pain between groups when the expected cell count was less than 5. The statistical significance for all tests was set as *p* < 0.05.

## Results

Due to drop-out and failure to complete the questionnaires, data from 99 (*n* = 102) participants were available for final analysis. Reasons given were difficulties with timings to attend appointments and athletes travelling. Table 1 summarizes the demographic characteristics of the whole population. A significant difference was shown, stratified by group for the age of the skiers 18.2 (SD 1.1) compared with the controls 16.4 (SD 0.6, *p* = 0.001). Comparison of gender stratified by group showed 47% of the skiers to be female and 53% male compared with the control group that showed 67% to be female and 33% to be male.

Table 2 shows the lifetime prevalence of back pain for the skiers (50%, *n* = 37) compared with the controls (44%, *n* = 11) (n.s.). A significant difference was shown for the greatest level of pain VAS 5.3 (SD 3.06) recorded during the past 6 months in the skiers compared with the controls VAS 2.4 (SD 1.98, *p* = 0.025). The greatest difference (Table 3) was shown in the skiers (46%, *n* = 17) for duration of back pain > 5 years compared with the controls (9%, *n* = 1) (n.s.).

**Table 2:** Lifetime prevalence, duration and onset of back and hip pain stratified by group

	Skiers (*n* = 74)	Controls (*n* = 25)	Total (*n* = 99)	*p* value
Back pain prevalence	37 (50%)	11 (44%)	48 (48%)	n.s.
Hip pain prevalence	16 (22%)	2 (8%)	18 (18%)	n.s.

Values are expressed as proportion and percentages in each category unless specified otherwise

**Table 3:** Duration and onset of back pain in years

	Skiers (*n* = 37)	Controls (*n* = 11)	*p* value
Duration of back pain			n.s.
6–12 months	4 (11%)	2 (18%)	
> 1 year	11 (30%)	2 (18%)	
> 5 years	17 (46%)	1 (9%)	
Onset of back pain (years)	14 (1.96)	13 (4.43)	n.s.

Duration of pain expressed as proportions and percentage in each category

For first episode values are years and standard deviation

The lifetime prevalence of hip pain (Table 2) for the skiers was (21%, *n* = 16) compared with the controls (8%, *n* = 2) (n.s.). Table 3 shows the values for the duration and onset of hip pain between groups, (58%, *n* = 7) skiers were shown to have a duration of hip pain > 1 year, with (29%, *n* = 4) first shown to report an onset of hip pain at 11–13 years of age, whilst the greatest number of skiers (42%, *n* = 6) reported an onset of 17–19 years of age.

There were no significant differences in Quality of life disability stratified by group for back pain using the EQ-5D with the skiers scoring 0.85 (SD 0.14) compared with the controls 0.88 (SD 0.13) (n.s.); this was similar for the ODI with the skiers scoring 9.4 (SD 8.36) compared with the controls 7.5 (SD 4.43) (n.s.).

## Discussion

The most important findings in the present study show that young Elite skiers do not have increased lifetime prevalence for back pain and hip pain compared with controls. Moreover, it could be suggested that one reason why 50% of young Elite skiers reported back pain may be in part related to the intense training and loading of the young athletic spine [3], which has been shown to be more vulnerable during growth-related spurts [1, 4]. Perhaps this was reflected with the VAS back pain scores between groups. A significant difference was shown for the skiers to have a greater level of back pain during the past 6 months VAS 5.3 compared with a VAS 2.4 for the controls (Table 4).

**Table 4:** Duration and onset of hip pain in years

	Skiers (%)	Controls (%)
Duration of hip pain		
6–12 months	17	0.0
> 1 year	58	50
> 5 years	17	50
Onset of hip pain (years)		
0–10 years	0.0	0.0
11–13 years	29	100
14–16 years	29	0.0
17–19 years	42	0.0

Duration of pain expressed as proportions and percentage in each category

**Fig. 1:**
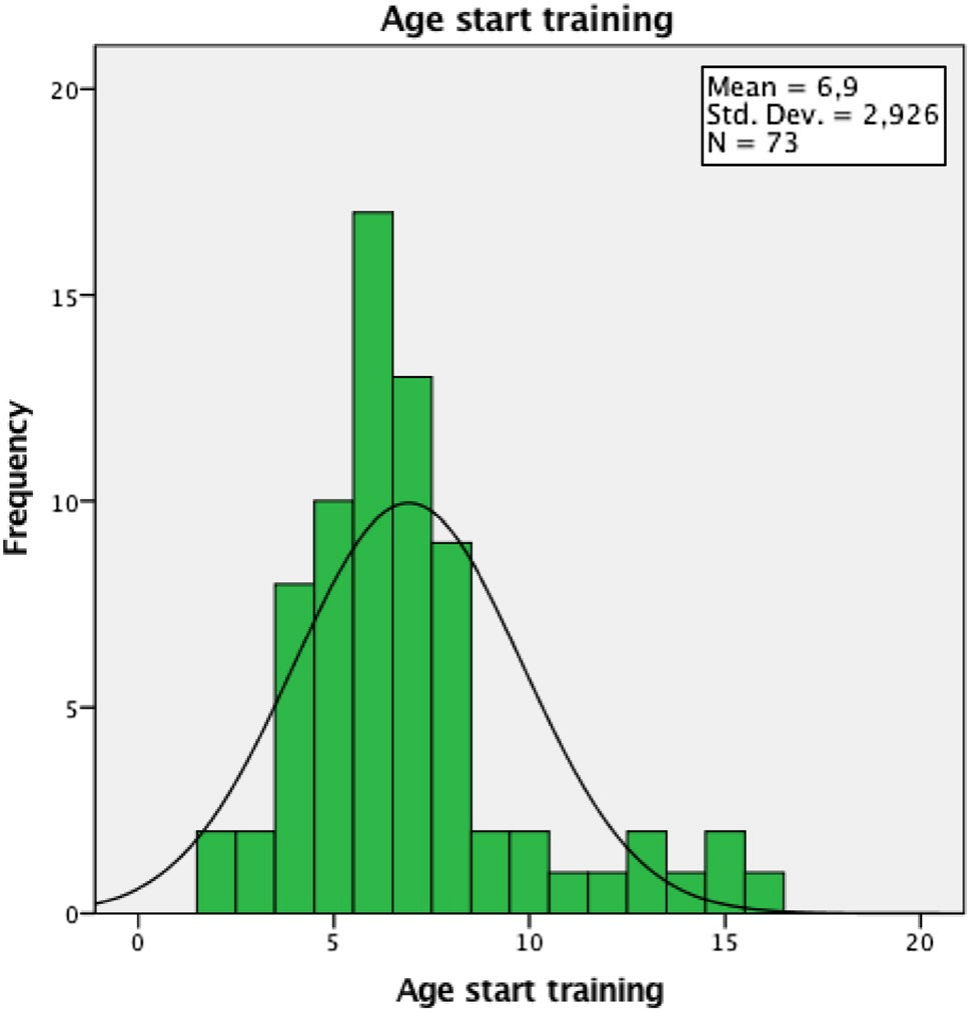
Histogram showing age of starting training for skiers

**Fig. 2:**
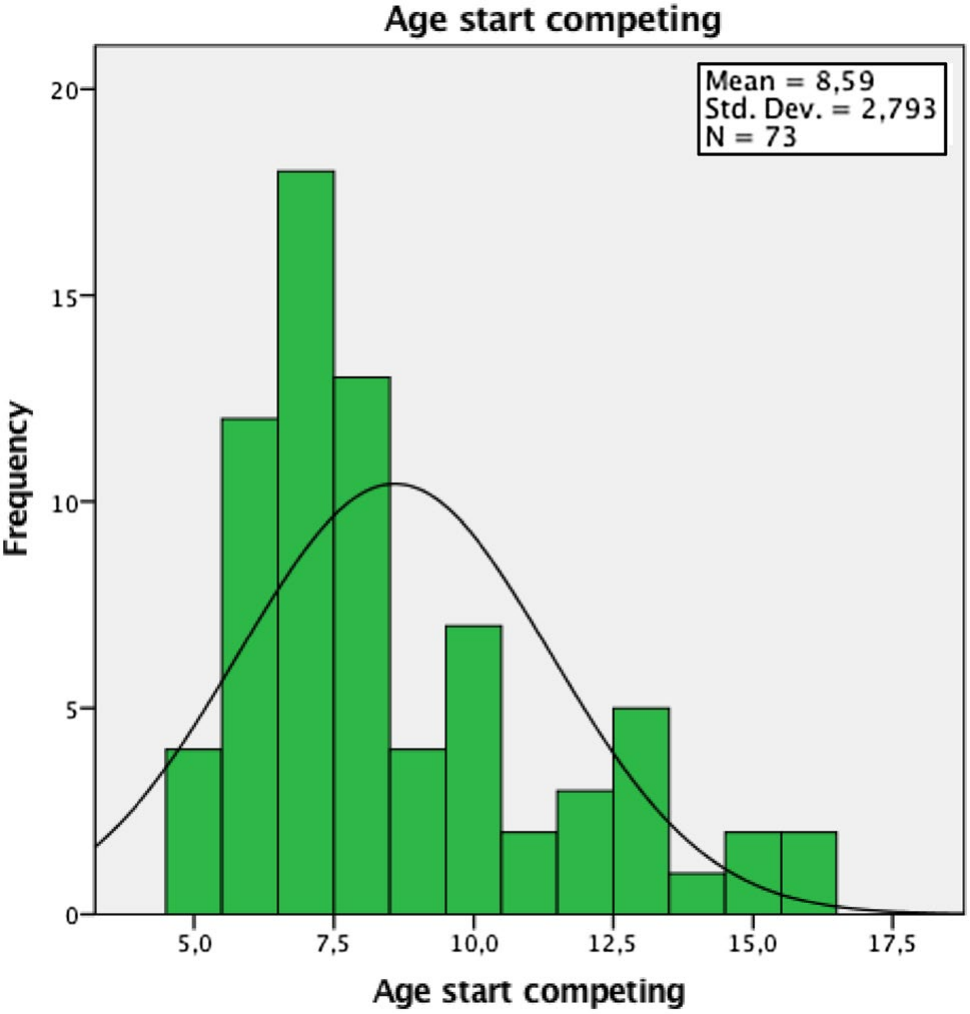
Histogram showing age of starting competition for skiers

The onset of back pain appeared similar between groups with the skiers reporting their first episode at 14 years and the controls at 13 years of age. This is in spite of the skiers beginning training at 6.9 years and competition at 8.6 years of age (Figs. 1, 2). Duration of back pain of > 5 years showed the greatest change to occur in the skiers (46%) compared with the controls (9%). This, however, must be viewed cautiously as missing values may have affected the outcome, as not all skiers completed their questionnaires properly, therefore biasing results. The clinical relevance of this may still suggest that a high number of skiers train and compete regularly with back pain and this may be a cause for concern in terms of the longevity of their athletic career. Previous studies by Thoreson et al. [36] and Peacock et al. [27] have shown similar results, but this is in conflict with an earlier study by Bergström et al. [7], which also included cross-country skiers and showed a higher prevalence of back pain in adolescent skiers (67%). Inclusion of cross-country skiers by Bergström et al. [7] may have affected their study outcome as different training regimes [3], different loading on the spine and hips and different skiing patterns may develop increasing variable degrees of spinal, knee and hip joint flexion for absorbing the effect of ground reaction forces [16, 21] (Table 5).

**Table 5:** Number of weekly training sessions for skiers

Training hours per week	Skiers (%)
3–5 h	6
6–8 h	21
9–11 h	25
> 11 h	38

Values are expressed as a proportion and percentage in each category

A higher lifetime prevalence of hip pain was shown in the skiers (22%) compared with the controls (8%), which appears similar to those studies previously reported in other sports [18, 26]. The onset of hip pain, for the skiers (29%), was first shown to have occurred between 11 and 13 years of age, which was similar for the controls, whilst the greatest percentage of hip pain was shown to occur in the skiers (43%) between the ages of 17–19 years. Unfortunately, this cannot be compared with the controls, as the mean age of the control group was 16.4 years. The clinical relevance of this might suggest that both young skiers and controls may have been subjected to hip joint growth-related disturbances [17, 31], which may have contributed to an increased prevalence of hip pain in their later teenage years. Increased hip joint morphology has previously been shown to occur in young elite athletes and non-athletes [17]. However, it could be hypothesized that a greater increment in the duration of hip pain shown in the skiers may have coincided with exercise loading intensities [3]. Previous studies have suggested training regimes, and early elite sports specialization may affect spinal alignment and spino-pelvic parameters [2, 37–39]; therefore, it would appear reasonable to hypothesize that such heavy loading may also impact the hip joint (Table 6).

**Table 6:** Lifetime prevalence of training hindrance for skiers

	Percentage (%) of back pain	Percentage (%) of hip pain
No	22	25
Once	19	19
2–10 times	29	38
> 10 times	22	6

Values are expressed as a percentage in each category

In the present study, a greater prevalence of radiating back pain was shown to occur with the skiers (24%) compared with the controls (9%). A specific LBP type such as radiating or referred pain has previously been suggested as a cause of spine and hip pain with one study showing that a lumbar spine disc herniation may refer distally into the hip joint [5]. Previous studies have shown a correlation to occur between back and hip pain resulting in such a coupled pain syndrome [5, 15, 40]. However, no such correlation was possible with the present study due to such a limited cohort and low numbers of participant’s actually reporting back and hip pain. The lifetime prevalence for back pain (29%) and hip pain (38%) due to training hindrance for the skiers appears similar to a previous study that reported (30%) absence from training [23]. It could be suggested that a greater number of skiers continue training whilst suffering from long-term back and hip pain and highlights the importance for the possibility of a long-term prevention study within this sporting discipline.

Both groups scored lower than 19% indicating minimal disability with the ODI and had a score of 1 or less for the EQ-5D questionnaires, indicating full health. A statistical difference was shown for the VAS; it should be highlighted that this is a subjective measuring tool that has shown to be a reliable and valid method designed to measure a change in pain, before and after a particular intervention [24, 29]. However, this could be viewed as a study limitation, as the VAS was not designed to measure pain levels between groups. Participants may have had difficulties with translating their subjective levels of back or hip pain to correspond with a particular point denoting a quantitative measurement on the scale [8]. It is possible that the skiers due to the nature of their training and competing may have developed an increased robustness, stronger personality traits and increased coping mechanisms to deal with pain. Errors may have occurred with interpreting and reproducing pain levels with the VAS scale, and this may have led to a bias between groups. The skiers due to having been subjected to longer periods of back and hip pain may have actually quantified higher levels of pain as moderate, and conversely the controls may have catastrophized their pain and quantified moderate and lower levels of pain as high.

Another study limitation was that the groups (skiers and controls) were not age-matched. The intentions of the present study were to include aged-matched groups; however, one reason for such a difference may be that the skiers were from grades 1–4 and the controls from the first grade at High School. Unfortunately, some skiers may have previously lived or studied abroad due to training and competition commitments and would have chosen to attend Åre High School because of the association with the Ski Academy. However, an important point must be highlighted, the present study was part of a larger investigation involving radiological examinations, and all participants were shown to have closed spinal physis on plain radiographs and closed hip joint physis on Magnetic Resonance Imaging (MRI), therefore limiting the possibility of growth-related spurts between groups.

There are other limitations to the present study. Selection of a larger control sample and performing a sample size calculation may have shown a greater prevalence for the values for back and hip pain between groups. No sample size calculation was performed as all the available participants from Åre High School that volunteered were included in the study. Recall bias, including interpreting and completing all the questionnaires correctly, may have affected the study. The inclusion criteria for the control group were to have exercised less than 2 h per week; therefore, the controls may have also been at risk of back pain due to reduced activity levels. Some of the young Elite skiers may have had premature career-ending injuries due to back and hip pain and disability; therefore, those participants may not have been included and biased the outcome.

The present study was able to show that young Elite skiers do not have increased lifetime prevalence for back pain and hip pain. Moreover, a high percentage of skiers (46%) reported duration of back pain prevalence > 5 years but this was not statistically significant. Therefore, the present study was unable to accept the hypothesis that there is increased lifetime prevalence for spine and hip pain in Elite skiers compared with a non-athletic control group.

## Conclusion

The potential impact of these findings may suggest many young Elite skiers continue to train and compete regularly with back pain or hip pain. Preventative measures should be taken with young Elite skiers to monitor training loads and intensities and to reduce or avoid high loads upon the spine and hip joints. This may in part help to reduce the risk of overload and repetitive injuries. Future studies should include a larger cohort with more sensitive, validated questionnaires to increase the possibility for a more in depth investigation for the lifetime prevalence and correlation of back and hip pain in young Elite skiers.

## Abbreviations

EQ 5D: Equality of life questionnaire
ODI: Oswestry Disability Index
LBP: Low back pain
MRI: Magnetic Resonance Imaging
VAS: Visual Analogue Scale
